# Contrast Enhancement Is Associated with a Higher DSC MRI-Derived Cerebral Metabolic Rate of Oxygen Index in Untreated Glioblastoma

**DOI:** 10.3390/diagnostics16091405

**Published:** 2026-05-06

**Authors:** Jonas Reis, Marco Öchsner, Chiara Adam, Thomas D. Fischer, Thomas Liebig, Robert Forbrig

**Affiliations:** Institute of Neuroradiology, University Hospital, LMU Munich, Marchioninistr. 15, 81377 Munich, Germany; jonas.reis@med.uni-muenchen.de (J.R.); marco.oechsner@med.uni-muenchen.de (M.Ö.); thomas.fischer@med.uni-muenchen.de (T.D.F.); thomas.liebig@med.uni-muenchen.de (T.L.)

**Keywords:** glioblastoma, dynamic susceptibility contrast MRI, CMRO_2_ index, contrast enhancement

## Abstract

**Background/Objectives:** Contrast enhancement (CE) on T1-weighted MRI is routinely used to guide therapy in the management of glioblastoma, although adjacent non-contrast-enhancing (non-CE) T2/FLAIR abnormalities can also harbor viable tumor tissue. The differences between these radiographic compartments remain incompletely characterized beyond conventional structural imaging. We therefore compared CE and non-CE compartments in untreated *IDH*-wildtype glioblastoma using dynamic susceptibility contrast (DSC) and diffusion-weighted MRI derived indices. **Methods**: Adults with untreated glioblastoma imaged preoperatively between January 2021 and September 2024 on multi-vendor 1.5 T and 3 T scanners were retrospectively included. Regions of interest were placed in CE tumor, adjacent non-CE T2/FLAIR hyperintense tissue, and contralateral normal-appearing white matter (NAWM). Mean apparent diffusion coefficient (rADC), cerebral blood volume (rCBV), capillary transit time heterogeneity (rCTH), oxygen extraction fraction (rOEF), and a cerebral metabolic rate of oxygen index (rCMRO_2_) were extracted and harmonized for scanner effects and normalized to NAWM. Paired CE–non-CE differences were tested using Wilcoxon signed-rank tests and summarized by Hodges–Lehmann differences with bootstrap 95% confidence intervals. Spearman correlations were used to assess coupling within contrast-enhancing tumor regions. **Results**: Seventy-two participants were analyzed (median age 67 years; 34 women); 66 had paired CE and non-CE data. rCMRO_2_ and rCBV were higher in CE than non-CE (both *p* < 0.001), while rADC was lower (*p* = 0.003). rOEF (*p* = 0.12) and rCTH (*p* = 0.52) did not differ significantly between compartments. **Conclusions**: CE in untreated *IDH*-wildtype glioblastoma predominantly reflects higher perfusion capacity (rCBV) along with a higher model-derived rCMRO_2_ index, while capillary-function indices (rCTH and rOEF) are not consistently compartment-restricted. These findings may refine the physiological interpretation of CE in glioblastoma and support further validation of DSC-derived indices.

## 1. Introduction

*IDH*-wildtype glioblastoma is defined by integrated histopathologic and molecular criteria according to the World Health Organization Classification of Tumors of the Central Nervous System (5th edition) [1]. In evidence-based treatment guidelines, MRI remains indispensable for delineating macroscopic tumor extent and for surgery and radiotherapy planning [2]. However, conventional structural MRI is an imperfect surrogate of tumor cellularity and biological activity. Biopsy-correlated studies have demonstrated substantial viable tumor burden in both contrast-enhancing (CE) and non-enhancing (non-CE) regions [3], even though the contrast-enhancing compartment is routinely operationalized as a target for surgery, radiotherapy, and response assessment and is therefore often treated as the dominant radiographic target and interpreted as the viable tumor core [4,5,6].

Physiologically, gadolinium enhancement arises when a blood–brain barrier (BBB) dysfunction permits extravasation and accumulation of contrast agent in the extravascular extracellular space; therefore, CE is most directly linked to BBB dysfunction and permeability changes and is not a specific marker of angiogenesis or viable tumor burden [7,8,9,10]. Dynamic susceptibility contrast (DSC) MRI is widely deployed for hemodynamic assessment in neuro-oncology [9,11], and recent consensus recommendations aim to improve standardization and reproducibility of DSC-derived biomarkers across sites and platforms [11,12,13,14]. While DSC-derived relative cerebral blood volume (rCBV) is an established marker of tumor vascularity [15,16,17], the DSC signal time course also contains information on microvascular flow patterns that influence oxygen extraction and metabolism [18,19,20,21]. Importantly for clinical translation, DSC-based capillary function modeling can estimate capillary transit time heterogeneity (CTH), which quantifies the dispersion of capillary transit times within the tissue and is a determinant of the maximum attainable oxygen extraction fraction (OEF) [9,22,23]. However, compartment-resolved comparisons remain limited and method-heterogeneous, highlighting ongoing uncertainty regarding compartmental perfusion physiology in untreated disease.

In this study, we primarily hypothesized that glioblastoma exhibits a distinct DSC-perfusion phenotype with different perfusion capacity (CBV), capillary function (OEF and CTH) and oxygen metabolism (CMRO_2_) indices, complemented by diffusivity (ADC), between CE and non-CE compartments in glioblastoma. Secondarily, we tested within-CE coupling and cross-compartment concordance of these indices to contextualize compartmental physiology.

## 2. Materials and Methods

### 2.1. Study Design and Cohort

We conducted a retrospective, single-center imaging study of consecutive adults with newly diagnosed, untreated *IDH*-wildtype glioblastoma (WHO grade 4). Patients were identified from the institutional database over a predefined accrual window (January 2021 to September 2024) and linked to the preoperative MRI archive. The inclusion criteria were as follows: (i) histopathologically confirmed *IDH*-wildtype glioblastoma according to the 2021 WHO CNS classification and (ii) preoperative MRI, including gradient-echo (GRE) DSC perfusion, diffusion-weighted imaging enabling ADC mapping, T2-weighted and/or fluid attenuated inversion recovery (FLAIR) imaging, and post-contrast T1-weighted imaging. Exclusion criteria were severe motion or susceptibility artifacts, incomplete or corrupted DSC data, prior therapy, or absence of a solid and sufficiently circumscribed enhancing component suitable for region of interest (ROI) placement. In total, 382 patients with newly diagnosed *IDH*-wildtype glioblastoma were screened, of whom 105 met the imaging inclusion criteria. Seventeen were excluded due to severe artifacts or incomplete/corrupted DSC data, and 16 were excluded because the tumor was non-enhancing, the enhancing component was not sufficiently circumscribed, or was too small to measure, yielding a final cohort of 72 patients. Demographic and clinical variables were extracted from clinical records. Generative artificial intelligence (GPT-5.2, OpenAI, San Francisco, CA, USA) was used to support language polishing and to assist with editing/debugging of Python 3.11.8 scripts used for data handling and analysis. All conceptualization, analyses, interpretation, and critical reasoning were performed by the authors, who take full responsibility for the final manuscript; no patient-identifiable or confidential information was entered into any artificial intelligence tool.

### 2.2. MRI Acquisition

Clinical MRI was performed as part of routine care using 1.5 T and 3 T scanners (Magnetom Sola Fit 1.5 T and Magnetom Vida 3 T, Siemens Healthineers, Erlangen, Germany; Signa HD 3 T, GE Healthcare, Chicago, IL, USA). GRE-DSC perfusion was performed using a standardized preload and bolus protocol. A preload of gadobutrol (0.05 mmol/kg) was administered to reduce T1 leakage effects, followed by a bolus of gadobutrol (0.1 mmol/kg) injected at 3–5 mL/s, followed by a 20 mL saline flush [14,15,24,25]. The DSC sequence parameters were scanner-specific and are listed in Appendix A Table A1. Structural sequences (T1 pre-/post-contrast, T2, FLAIR) and diffusion-weighted imaging were acquired according to the routine preoperative tumor protocol.

### 2.3. Image Post-Processing and Perfusion Parameter Maps

DSC data were post-processed using a vendor-neutral software (Neurosuite v16.1, Cercare Medical, Aarhus, Denmark). The software performs automated arterial input function (AIF) identification, standard AIF-coupled leakage correction, motion correction, and deconvolution-based residue function estimation. The pipeline generated maps of CBV, CTH, OEF, and CMRO_2_. All capillary function and oxygen metabolism outputs were interpreted as method- and model-specific MRI-derived indices rather than absolute PET-equivalent quantities. A description of the model-specific definitions for CTH, OEF, and CMRO_2_ is provided in Appendix A. All parametric maps were co-registered to the anatomical images before ROI extraction.

### 2.4. Region of Interest Definition

A single board-certified neuroradiologist with 7 years of experience in neuro-oncologic imaging placed all ROIs. To minimize bias, the reader was blinded to ADC and DSC-derived parametric maps during ROI placement and used only structural sequences for guidance. Three circular two-dimensional ROIs of approximately 50 mm^2^ each were placed in each of three tissue classes: (i) solid contrast-enhancing tumor (CE), selected from visually representative enhancing tissue on post-contrast T1-weighted imaging, (ii) non-enhancing tumor-related tissue (non-CE), defined operationally as T2/FLAIR-hyperintense tissue, immediately adjacent to the enhancing margin and within approximately 20 mm of the outer enhancing rim, and (iii) contralateral NAWM without T2/FLAIR hyperintensity. ROIs were positioned to avoid macroscopic necrosis, hemorrhage, cortical gray matter, visible macro vessels, cerebrospinal fluid, and areas with marked susceptibility or motion artifacts. When multiple anatomically valid candidate regions were available, three non-overlapping ROIs were placed to sample representative tissue while maximizing spatial separation within the respective compartment. For each ROI, mean voxel values were extracted from ADC, CBV, CTH, OEF, and CMRO_2_ maps after co-registration. Within each tissue class, after harmonization, the three ROI means were averaged to yield one subject-level value per metric and tissue compartment for subsequent normalization and statistical analyses.

### 2.5. Harmonization and Normalization

To mitigate scanner- and field-strength-related effects, the subject-level tissue means were harmonized using empirical Bayes ComBat harmonization (location only), with batch defined as vendor × field strength [25,26,27]. Location-only harmonization was used to reduce batch-related mean shifts while avoiding additional scale adjustment in the setting of unbalanced vendor/field-strength groups. No biological or clinical covariates were included in the ComBat model because the analysis focused on within-subject compartmental contrasts within a single disease entity. ComBat was applied in a transformed domain using monotone transforms appropriate for each metric: a natural logarithm for strictly positive variables (ADC, CBV, CTH, and CMRO_2_) and a logit transform for OEF. Harmonized values were then back-transformed to native units.

For primary analyses, all metrics were expressed as relative NAWM-normalized ratios: rMetric(CE) = Metric(CE)/Metric(NAWM) and rMetric(non-CE) = Metric(non-CE)/Metric(NAWM) for ADC, CBV, CTH, OEF, and CMRO_2_. Harmonization performance was evaluated using the proportion of NAWM variance explained by the batch (one-way ANOVA R^2^) before and after ComBat in the transformed domain (Appendix A Table A2).

### 2.6. Statistical Analysis

Continuous variables are summarized as medians (interquartile ranges). The primary analysis comprised the within-subject paired difference in mean rCBV, rCTH, rOEF, rCMRO_2_, and rADC between CE and non-CE tissue using Wilcoxon signed-rank tests. Effect sizes were summarized using Hodges–Lehmann estimates of the median paired difference with 95% confidence intervals obtained by percentile bootstrapping (4000 resamples). Multiplicity across the five paired compartment tests was controlled using the Benjamini–Hochberg false discovery rate (BH-FDR).

Secondary analyses assessed physiologic coupling within CE regions using Spearman rank correlations between rOEF(CE) and rCTH(CE), and between rCMRO_2_(CE) and rCTH(CE), with bootstrap confidence intervals (4000 resamples) and two-sided permutation *p*-values (3000 permutations). A partial Spearman correlation between rCMRO_2_(CE) and rCTH(CE) controlling for rCBV(CE) was computed using rank residualization with permutation inference analysis. To characterize whether each metric behaved more as a within-patient phenotype versus a compartment-localized feature, cross-compartment concordance was assessed by correlating the CE and non-CE regions for each metric (Spearman; pairwise complete cases) using bootstrap confidence intervals and BH-FDR correction across the five concordance tests. Sensitivity analyses without NAWM normalization were performed using absolute ComBat-harmonized metrics (Appendix A Table A3). All statistical tests were two-sided. An adjusted q-value < 0.05 after BH-FDR correction was considered statistically significant for families of tests where FDR correction was applied; otherwise, *p* < 0.05 was considered statistically significant. Missing data were handled by pairwise deletion; no imputation was performed. Statistical analyses were performed using Python 3.11.8 (NumPy 1.24.0, pandas 1.5.3, SciPy 1.14.1, Matplotlib 3.6.3, Statsmodels 0.13.5).

## 3. Results

### 3.1. Cohort and Harmonization

**Figure 1:**
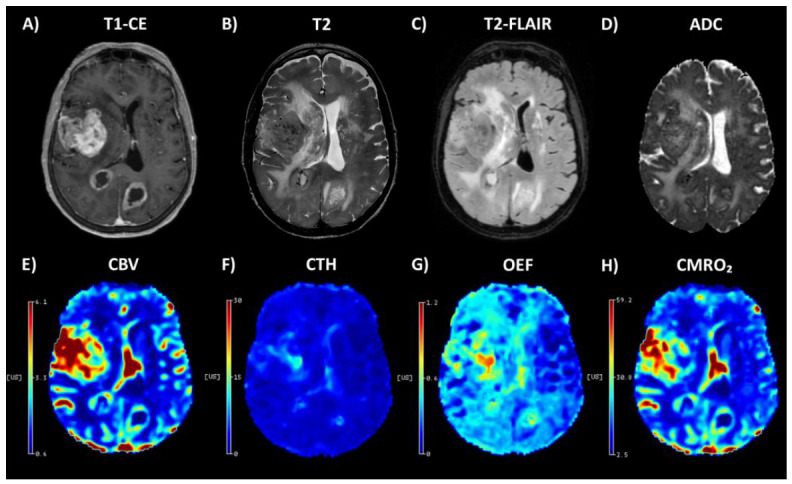
Representative preoperative MRI at 3 T with DSC-derived parametric maps in untreated *IDH*-wildtype glioblastoma (MGMT promoter unmethylated, TERT promoter wildtype; 3 T). (**A**) Post-contrast T1-weighted imaging (T1-CE), (**B**) T2-weighted imaging, (**C**) T2-FLAIR, (**D**) apparent diffusion coefficient (ADC) map, (**E**) cerebral blood volume (CBV) map, (**F**) capillary transit time heterogeneity (CTH) map, (**G**) oxygen extraction fraction (OEF) map, and (**H**) cerebral metabolic rate of oxygen (CMRO_2_) index map. Color bars indicate the relative scale of the map.

The clinical, molecular, and baseline imaging characteristics of the cohort (*n* = 72) are summarized in Table 1, and an illustrative case is shown in Figure 1. Paired CE–non-CE analyses were performed on 66 complete pairs for each metric, whereas CE-based analyses that did not require non-CE retained all subjects with available CE and NAWM values (*n* = 72). ComBat harmonization reduced batch-associated variance in NAWM metrics across all parameters (Appendix A, Table A2), consistent with the effective mitigation of vendor and field-strength effects.

### 3.2. Within-Subject Paired Comparisons Between CE and Non-CE Regions

**Figure 2:**
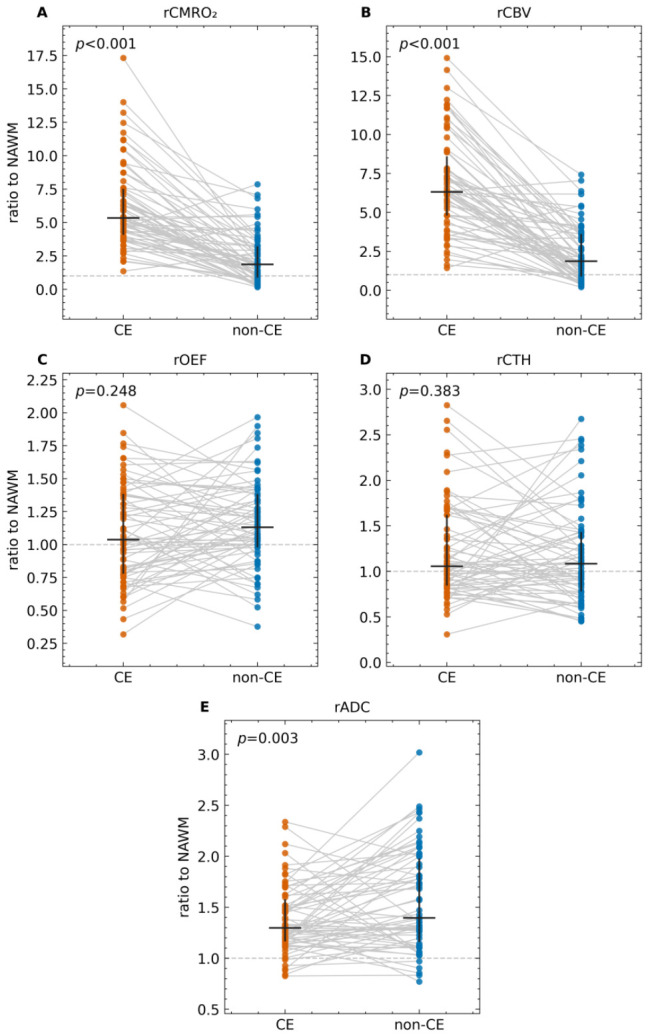
Paired CE versus non-CE differences in NAWM-normalized indices for rCMRO_2_ (**A**), rCBV (**B**), rOEF (**C**), rCTH (**D**), and rADC (**E**), depicting significant differences between compartments for rCMRO_2_, rCBV and rADC. Each line connects paired CE and non-CE values within a patient; points denote individual patients. Vertical bars indicate median and interquartile range (IQR). The dashed horizontal line marks ratio = 1.0 (equal to NAWM). Two-sided Wilcoxon signed-rank tests were used for inference. Abbreviations: CE, contrast-enhancing; non-CE, non-contrast-enhancing; NAWM, normal-appearing white matter; rADC, relative apparent diffusion coefficient; rCBV, relative cerebral blood volume; rCTH, relative capillary transit time heterogeneity; rOEF, relative oxygen extraction fraction; rCMRO_2_, relative cerebral metabolic rate of oxygen.

The results of paired compartmental comparisons of NAWM-normalized ratios (in CE vs. non-CE regions) are displayed in Table 2. rCMRO_2_ was significantly higher in CE than in non-CE (Wilcoxon *p* < 0.001, q < 0.001; Figure 2A). Across the same paired tests (BH-FDR across five metrics; *n* = 66), CE regions also demonstrated significantly higher rCBV than non-CE regions (*p* < 0.001, q < 0.001; Figure 2B) and significantly lower rADC (*p* = 0.003, q = 0.006; Figure 2E) than in non-CE regions. In contrast, mean values for rOEF (*p* = 0.12, q = 0.15; Figure 2C) and rCTH (*p* = 0.52, q = 0.52; Figure 2D) did not significantly differ between CE and non-CE regions.

### 3.3. CE Coupling Analyses

**Figure A1:**
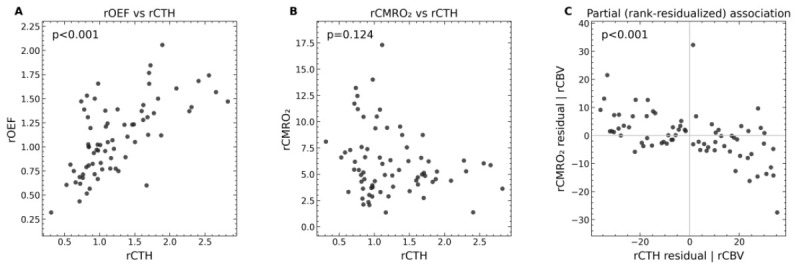
Coupling of capillary function and oxygen metabolism indices in CE. (**A**) rOEF versus rCTH, (**B**) rCMRO_2_ versus rCTH, and (**C**) residualized (rank-based) association between rCMRO_2_ and rCTH, controlling for rCBV as a pragmatic perfusion-capacity surrogate. Abbreviations: CE, contrast-enhancing; rCBV, relative cerebral blood volume; rCTH, relative capillary transit time heterogeneity; rOEF, relative oxygen extraction fraction; rCMRO_2_, relative cerebral metabolic rate of oxygen.

Table 3 comprises the results of the CE coupling analyses (*n* = 72). Within the CE compartment, rOEF and rCTH showed a strong positive correlation (Spearman ρ = 0.707; *p* < 0.001, q < 0.001; permutation *p* < 0.001; Figure A1A). rCMRO_2_ was modestly inversely correlated with rCTH (ρ = −0.245; *p* = 0.044, q = 0.038; permutation *p* = 0.044; Figure A1B). This inverse association was strengthened in the prespecified partial Spearman analysis controlling for rCBV as a proxy for perfusion capacity (ρ_partial_ = −0.531; permutation *p* < 0.001; Figure A1C).

### 3.4. CE-Non-CE Concordance and Sensitivity Analyses

To assess the degree to which each metric behaved as a patient-level trait versus a compartment-specific feature, we quantified CE–non-CE concordance using NAWM-normalized ratios (pairwise complete cases; *n* = 66, Table 4). The CE–non-CE concordance was not significant for rCMRO_2_ (ρ = 0.201, *p* = 0.11, q = 0.13) and rCBV (ρ = 0.178, *p* = 0.15, q = 0.15). Modest but significant CE–non-CE concordance was observed for rOEF (ρ = 0.341, *p* = 0.005, q = 0.009), rCTH (ρ = 0.338, *p* = 0.006, q = 0.009) and rADC (ρ = 0.397, *p* < 0.001, q = 0.005). These results were directionally consistent in sensitivity analyses without NAWM normalization (Appendix A Table A3).

## 4. Discussion

In this cohort of adults with untreated *IDH*-wildtype glioblastoma, contrast enhancement was associated with a compartment characterized by a higher model-derived rCMRO_2_ index, higher perfusion capacity (rCBV), and lower diffusivity (rADC; consistent with higher cellularity) compared with adjacent non-enhancing T2/FLAIR abnormality. In contrast, the capillary function indices rOEF and rCTH did not significantly differ between compartments. These findings refine the physiological interpretation of contrast enhancement: CE appears to identify a peak in vascular/perfusion capacity and CMRO_2_ index but does not necessarily imply higher mean oxygen extraction efficiency or a distinct shift in capillary-function indices.

However, the interpretation of the rCMRO_2_ index requires careful consideration. In the present workflow, rCMRO_2_ is a model-derived, method-specific measure that is proportional to perfusion capacity and rOEF, assuming an approximately constant arterial oxygen content. We did not analyze rCBF because its estimates are comparatively sensitive to AIF selection and deconvolution choices in routine clinical DSC pipelines [28], whereas rCBV is the most widely standardized DSC biomarker in neuro-oncology [12,14]. Therefore, we treated rCBV as a pragmatic surrogate of perfusion capacity when interpreting rCMRO_2_, acknowledging that rCBV is not equivalent to rCBF when the mean transit time varies [9,21].

The CE–non-CE differences in rCBV and rADC reproduce established perfusion and diffusion signatures of enhancing glioblastoma and align with the clinical use of DSC MRI to characterize tumor vascularity [12,14,29,30]. The incremental contribution of the present study lies in the compartment-resolved behavior of DSC-derived oxygen-related model outputs. First, rCMRO_2_ directionally paralleled the CE vascular/perfusion-capacity phenotype, whereas the capillary-function indices (rOEF and rCTH) did not show a corresponding mean compartmental shift. Second, within CE, the inverse association between rCMRO_2_ and rCTH strengthened after accounting for rCBV, suggesting that capillary transit-time heterogeneity may capture microvascular information that is not summarized by vascular volume alone. Third, cross-compartment concordance differed by metric: rADC, rOEF, and rCTH showed greater within-patient coherence across compartments, whereas rCBV and rCMRO_2_ appeared more compartment-dependent. Together, these findings suggest that the CMRO_2_ index and capillary-function indices provide complementary, rather than interchangeable, physiologic information beyond established rCBV and ADC [9,14].

The harmonization strategy also supports the translational framing of this study. ComBat reduced vendor/field-strength-associated variance in NAWM across all metrics, indicating that the workflow can mitigate major scanner-related mean shifts in routine multi-vendor data. Nevertheless, harmonization cannot fully remove protocol-dependent bias or substitute for prospectively standardized multicenter acquisition.

In contrast to rCBV and rCMRO_2_, rOEF and rCTH did not differ significantly between CE and non-CE at the ROI-mean level. However, paired CE–non-CE values for rOEF and rCTH showed greater dispersion and more frequent direction reversals across individuals than rCBV and rCMRO_2_, indicating that capillary function indices are less consistently compartment-locked in this cohort. This pattern is plausible because non-enhancing T2/FLAIR abnormalities surrounding glioblastoma are heterogeneous and may include infiltrative tumor intermingled with edema and reactive microenvironmental changes, rather than representing a purely benign comparator [3,31]. The absence of a robust CE–non-CE shift in rOEF and rCTH should therefore not be interpreted as evidence of equivalence, because no equivalence testing was performed. Rather, the paired estimates indicate no robust ROI-mean compartmental shift, while smaller, spatially heterogeneous, or voxel-level differences may remain undetected. From a statistical power perspective, the paired sample was sufficient to detect the large rCBV and rCMRO_2_ effects observed here, but it was not designed to exclude smaller compartmental effects in rOEF or rCTH, particularly given their greater within-subject dispersion and model-derived variance. Supporting this interpretation, cross-compartment concordance analyses showed significant within-patient coherence for rADC, rOEF, and rCTH across CE and non-CE tissue, whereas concordance was not significant for rCBV and rCMRO_2_. Translationally, these findings suggest that diffusion and capillary function indices may reflect broader within-patient tumor properties that span radiographic compartments, whereas perfusion capacity and CMRO_2_ index peaks appear more spatially concentrated within enhancement and therefore depend more strongly on where within the lesion they are sampled.

OEF and CTH are co-estimated within the same modeling framework, and their correlation may reflect both shared physiological determinants and shared estimation structure rather than independent biomarker behavior [20]. Therefore, we interpret this coupling primarily as indicative of physiological and model consistency within the enhancing compartment. In parallel, rCMRO_2_(CE) showed an inverse association with rCTH(CE), which strengthened after accounting for rCBV(CE) as a proxy for perfusion capacity. This conditional relationship is compatible with the concept of capillary dysfunction: greater transit-time heterogeneity may reflect microvascular inefficiency and functional shunting that limits effective oxygen utilization even in hypervascular tissue [21,22,23]. Although the present design does not support causal inference, the strengthened association after conditioning on rCBV suggests that microvascular heterogeneity may represent a physiological dimension not captured by vascular volume alone [19,32].

Our results complement previous oxygen metabolism mapping studies that used non-DSC techniques. A feasibility study using quantitative susceptibility mapping combined with quantitative blood oxygenation level dependent imaging and arterial spin labelling reported no significant differences between enhancing tumors and non-enhancing T2/FLAIR regions for CBF, OEF and CMRO_2_ in a small heterogenous cohort [33]. The present findings are compatible with limited compartmental separation for capillary function indices, while suggesting a robust CE–non-CE difference in a DSC-derived rCMRO_2_ in a larger, harmonized cohort study. Differences in methodology, modeling assumptions, and sample size preclude direct quantitative comparison.

Clinically, these findings should be viewed as hypothesis-generating. They suggest that routine GRE-DSC data may provide physiologic context for interpreting CE and adjacent non-CE tissue, with CE marking a vascular/perfusion-capacity and CMRO_2_-index peak rather than a complete surrogate for all oxygen-related physiology. If prospectively validated, compartment-resolved rCBV/rCMRO_2_ patterns could help inform biopsy or surgical sampling, radiotherapy target interpretation, and response assessment when enhancement alone is biologically ambiguous.

This study has several limitations. First, the retrospective single-center design may introduce selection bias and residual protocol heterogeneity despite harmonization. In particular, exclusion of non-enhancing tumors or tumors with insufficiently circumscribed enhancement biases the cohort toward lesions suitable for CE–non-CE ROI comparison. Second, rCTH, rOEF, and rCMRO_2_ are model-derived and method-dependent; they should be interpreted as relative DSC-derived indices rather than absolute PET-equivalent measures, and the underlying modeling assumptions may not hold uniformly across all glioblastoma microenvironments. Third, rCBF and rMTT were not reported; although rCBV provides a pragmatic surrogate of perfusion capacity, rCBV is not equivalent to flow when transit time varies. Fourth, ROI-mean sampling does not capture full volumetric heterogeneity and is susceptible to partial-volume and mixed-tissue effects, particularly in non-enhancing T2/FLAIR abnormality. ROI placement was performed by a single experienced reader, and inter-reader reproducibility was not assessed. Automated, segmentation-based volumetric compartmentation using separable CE and non-CE/edema component volumes may facilitate voxel-wise and longitudinal validation with reduced operator dependence [34,35]. Finally, the absence of significant rOEF and rCTH differences should not be interpreted as equivalence; smaller, spatially heterogeneous, or voxel-level differences may remain undetected at the ROI-mean level. The lack of external reference standards, such as [^15^O]-gas PET, tissue hypoxia markers, or spatially co-registered histology, limit biological specificity and causal interpretation.

Future studies should incorporate explicit rCBF and rMTT reporting, voxel-wise and volumetric compartment mapping, reader-reproducibility assessment, and independent reference standards to validate DSC-derived capillary-function and CMRO_2_ indices. Prospective multicenter designs with standardized DSC acquisition, harmonized post-processing, and longitudinal outcome data will be important to determine whether these compartmental phenotypes add incremental value beyond established perfusion and diffusion biomarkers for treatment planning and response assessment.

## 5. Conclusions

Contrast-enhancing glioblastoma regions were associated with a distinct vascular/perfusion capacity and model-derived CMRO_2_ index phenotype compared with adjacent non-enhancing T2/FLAIR abnormality. In contrast, the capillary function indices rOEF and rCTH did not differ significantly between compartments. These results refine the physiologic interpretation of enhancement by indicating that CE primarily marks a vascular/perfusion capacity and CMRO_2_ index peak rather than a distinct shift in capillary function indices. Future prospective studies should use independent reference standards to validate the model-derived rCMRO_2_ index and determine whether these compartmental phenotypes provide incremental value for treatment planning or response assessment.

## Figures and Tables

**Table 1:** Cohort characteristics and acquisition context.

Characteristic	Value
Total cohort, *n*	72
Age at scan, years	66.8 [58.3–76.9]
Sex, female	34 (47.2%)
MGMT promoter methylation, methylated	26 (36.1%)
MGMT promoter methylation, partially/unmethylated	46 (63.9%)
TERT promoter mutation (C228T/C250T)	60 (83.3%)
TERT wild-type	10 (13.9%)
TERT unknown	2 (2.8%)
Scanner vendor: Siemens	35 (48.6%)
Scanner vendor: GE	37 (51.4%)
Field strength: 3.0 T	61 (84.7%)
Field strength: 1.5 T	11 (15.3%)
Vendor × field: Siemens 1.5 T	11
Vendor × field: Siemens 3.0 T	24
Vendor × field: GE 3.0 T	37

Median values with IQR for continuous variables, as well as n (%) for categorical variables, are reported. Vendor and field-strength distributions are reported as counts. Key analytic sample sizes are provided, including the number of patients with paired CE–non-CE data available for compartment comparisons and the number contributing to CE-only coupling analyses. Abbreviations: CE, contrast-enhancing; non-CE, non-contrast-enhancing; NAWM, normal-appearing white matter; MGMT, O6-methylguanine-DNA methyltransferase; T. Tesla; TERT, telomerase reverse transcriptase; IQR, interquartile range.

**Table 2:** Primary and secondary paired CE versus non-CE differences in NAWM-normalized indices (*n* = 66).

Metric	CE Median [Q1–Q3]	Non-CE Median [Q1–Q3]	HL Δ (CE-non-CE)	95% CI	Wilcoxon *p*	BH-FDR q
rCMRO_2_	5.31 [4.12–7.63]	1.64 [0.81–3.01]	3.50	[2.94, 4.67]	<0.001	<0.001
rCBV	6.35 [4.64–8.54]	1.83 [0.86–3.21]	4.81	[3.32, 5.80]	<0.001	<0.001
rADC	1.30 [1.18–1.54]	1.41 [1.15–1.90]	−0.12	[−0.25, −0.01]	0.003	0.006
rOEF	1.00 [0.78–1.36]	1.14 [0.98–1.38]	−0.09	[−0.18, 0.04]	0.120	0.150
rCTH	1.08 [0.85–1.59]	1.08 [0.77–1.44]	0.04	[−0.06, 0.21]	0.521	0.521

Values are reported as median [Q1–Q3] for CE and non-CE. The paired effect is summarized by the Hodges–Lehmann estimate of the median paired difference (Δ = CE − non-CE) with 95% bootstrap confidence intervals. Two-sided Wilcoxon signed-rank tests were performed, with Benjamini–Hochberg false discovery rate correction across the five prespecified paired tests. Abbreviations: CE, contrast-enhancing; non-CE, non-contrast-enhancing; NAWM, normal-appearing white matter; rADC, relative apparent diffusion coefficient; rCBV, relative cerebral blood volume; rCTH, relative capillary transit time heterogeneity; rOEF, relative oxygen extraction fraction; rCMRO_2_, relative cerebral metabolic rate of oxygen; BH-FDR, Benjamini–Hochberg false discovery rate.

**Table 3:** Spearman correlations among CE capillary function and oxygen metabolism indices (*n* = 72).

Analysis	Spearman ρ	95% CI	Permutation *p*	BH-FDR q
rOEF vs. rCTH	0.71	[0.55, 0.84]	<0.001	<0.001
rCMRO_2_ vs. rCTH	−0.24	[−0.43, −0.04]	0.044	0.038
partial: rCMRO_2_ vs. rCTH|rCBV	−0.53	—	<0.001	—

Spearman correlations of rOEF versus rCTH and rCMRO_2_ versus rCTH. Partial Spearman correlation for rCMRO_2_ versus rCTH controlling for rCBV is reported using rank residualization with permutation inference. Bootstrap 95% confidence intervals and BH-FDR q-values are reported. CE, contrast-enhancing; NAWM, normal-appearing white matter; rADC, relative apparent diffusion coefficient; rCBV, relative cerebral blood volume; rCTH, relative capillary transit time heterogeneity; rOEF, relative oxygen extraction fraction; rCMRO_2_, relative cerebral metabolic rate of oxygen; BH-FDR, Benjamini–Hochberg false discovery rate.

**Table 4:** Spearman correlations between CE and non-CE for NAWM-normalized ratios of indices.

Analysis	Spearman ρ	95% CI	Permutation *p*	BH-FDR q
rCMRO_2_	0.20	[−0.04, 0.42]	0.103	0.132
rCBV	0.18	[−0.07, 0.40]	0.141	0.152
rADC	0.40	[0.17, 0.59]	<0.001	0.005
rOEF	0.34	[0.11, 0.55]	0.006	0.009
rCTH	0.34	[0.10, 0.55]	0.009	0.009

Bootstrap 95% confidence intervals and BH-FDR q-values are reported. Abbreviations: CE, contrast-enhancing; non-CE, non-contrast-enhancing; NAWM, normal-appearing white matter; rADC, relative apparent diffusion coefficient; rCBV, relative cerebral blood volume; rCTH, relative capillary transit time heterogeneity; rOEF, relative oxygen extraction fraction; rCMRO_2_, relative cerebral metabolic rate of oxygen; BH-FDR, Benjamini–Hochberg false discovery rate.

## Data Availability

The data reported in this article can be shared in compliance with current data protection regulations by the European Union and approval from the relevant regulatory authorities. All proposals should be directed to the corresponding author.

## Abbreviations

The following abbreviations are used in this manuscript:

ADC	Apparent diffusion coefficient
AIF	Arterial input function
ANOVA	Analysis of variance
ASL	Arterial spin labeling
BBB	Blood–brain barrier
BH-FDR	Benjamini–Hochberg false discovery rate
CBF	Cerebral blood flow
CBV	Cerebral blood volume
CE	Contrast-enhancing (tumor compartment)
CI	Confidence interval
CMRO_2_	Cerebral metabolic rate of oxygen (index)
CNS	Central nervous system
CTH	Capillary transit time heterogeneity
DCE	Dynamic contrast-enhanced
DSC	Dynamic susceptibility contrast
DWI	Diffusion-weighted imaging
EANO	European Association of Neuro-Oncology
ESTRO	European Society for Radiotherapy and Oncology
FLAIR	Fluid-attenuated inversion recovery
GBM	Glioblastoma
GRE	Gradient echo
HL	Hodges–Lehmann (median paired difference estimate)
IDH	Isocitrate dehydrogenase
IQR	Interquartile range
MRI	Magnetic resonance imaging
MTT	Mean transit time
NAWM	Normal-appearing white matter
non-CE	Non-enhancing (tumor-related T2/FLAIR abnormality compartment)
OEF	Oxygen extraction fraction
PET	Positron emission tomography
qBOLD	Quantitative blood oxygenation level dependent imaging
QIBA	Quantitative Imaging Biomarkers Alliance
QSM	Quantitative susceptibility mapping
r	Prefix indicating normalization to contralateral NAWM
RANO	Response Assessment in Neuro-Oncology
ROI	Region of interest
R^2^	Coefficient of determination
TE	Echo time
TR	Repetition time
WHO	World Health Organization

## Appendix A. Definition of Perfusion-Model Derived Indices

CTH was defined as the dispersion (standard deviation) of the fitted capillary transit time distribution. OEF was computed by integrating a single-capillary extraction model over the estimated transit time distribution. Neurosuite also outputs a model-based cerebral metabolic rate of oxygen (CMRO_2_) index. In the underlying model, this index is proportional to the product of tissue perfusion and OEF (CMRO_2_ index ∝ CBF × OEF) under the assumption of approximately constant arterial oxygen content. Because CBV is the most widely standardized DSC perfusion biomarker in neuro-oncology, and because DSC-derived CBF is comparatively sensitive to AIF and deconvolution choices, CBF was not analyzed as a standalone reported endpoint in this manuscript. Given that CBV and CBF are linked by the central volume relationship (CBV = CBF × MTT), CBV was treated as a pragmatic surrogate of perfusion capacity when interpreting throughput effects in this study. Accordingly, we report CBV alongside the CMRO_2_ index, and CBV was used as a proxy for the perfusion term in conditional analyses.

**Table A1:** GRE-DSC perfusion acquisition parameters.

Scanner	TR (ms)	TE (ms)	Flip Angle (°)	Temporal Resolution (s)
GE Signa HD	1400	20	60	1.40
Siemens Magnetom Vida	1600	30	90	1.60
Siemens Magnetom Sola fit	2770	31	90	2.77

Abbreviations: TR, repetition time; TE, echo time; GRE-DSC, gradient-echo dynamic susceptibility contrast.

**Table A2:** NAWM batch variance before and after ComBat.

Metric	R^2^ Pre-ComBat	R^2^ Post-ComBat
ADC	0.235	0.041
CBV	0.152	0.017
CTH	0.184	0.014
OEF	0.021	0.001
CMRO_2_	0.129	0.025

Batch-associated variance in NAWM for each metric quantified as one-way ANOVA R^2^ by vendor × field-strength batch before and after location-only ComBat harmonization in the transformed domain. Abbreviations: NAWM, normal-appearing white matter; ANOVA, analysis of variance; R^2^, coefficient of determination.

**Table A3:** Paired CE versus non-CE differences without NAWM normalization (*n* = 66).

Metric	Scale	HL Δ (CE–nonCE)	95% CI	Wilcoxon *p*	BH-FDR q
ADC	log(abs)	−0.088	[−0.218, −0.010]	0.005	0.009
CBV	log(abs)	1.208	[1.047, 1.537]	<0.001	<0.001
CTH	log(abs)	0.039	[−0.074, 0.212]	0.376	0.376
OEF	logit(abs)	−0.157	[−0.313, 0.050]	0.095	0.119
CMRO_2_	log(abs)	1.159	[1.010, 1.401]	<0.001	<0.001

Sensitivity analysis repeating paired CE versus nonCE comparisons using absolute (ComBat-harmonized, transformed-domain) metrics without NAWM ratio normalization. Effect sizes are reported as Hodges–Lehmann paired differences with 95% bootstrap confidence intervals; two-sided Wilcoxon signed-rank *p*-values and BH-FDR q-values across the five paired tests are provided. Abbreviations: CE, contrast-enhancing; nonCE, non-contrast-enhancing; BH-FDR, Benjamini–Hochberg false discovery rate.
